# Ganglion

**Published:** 1908-11-14

**Authors:** 


### Surgery.

### GANGLION.

A simple ganglion, so called, is a condition which is seen often enough in the out-patient department of a large hospital. It consists of a rounded swelling of varying size, situated in the line of a tendon. It is most commonly found on the back of the wrist, or on the dorsum of the foot, being then connected with one of the extensor tendons in either case. But it is also met with on the front of the wrist or at the outer side of the ankle joint in connection with one or other of the peronei tendons.

In an ordinary case the diagnosis is simple. The swelling gives a sensation of elasticity to the finger. It may appear to fluctuate if it is large. A more common abnormality, however, is that the swelling is very tense, and may then feel so hard that it is mistaken for an exostosis. It is not attached to the overlying skin, and it moves on the deep structures. But if the tendon with which it is connected is contracted, its mobility is immediately impaired. The characters of the swelling therefore should make its recognition an easy matter. Much difficulty may, however, be experienced in the case of a tense ganglion in an unusual situation. The writer saw such a case recently. The patient complained of a densely hard oval swelling about the size of a pigeon's egg below the inner tuberosity of the left knee. It had grown gradually and was painless. It did not move on the bone. This was taken to be an osteoma of the tibia. An incision was made preparatory to chiselling off the tumour; but, as soon as it was exposed, it was seen to be a tense ganglion in connection with the tendon of insertion of the sartorius. Its lack of mobility was explained by the fact that the sartorius is more or less closely bound down to the bone in this situation.

A ganglion ordinarily causes no symptoms, the patient merely complaining of the presence of an abnormal swelling; but if it occurs on the foot it may be pressed upon and irritated by the boot and so become painful.

Much ingenuity has been expended in suggesting different methods of causation of this condition; but it is accepted by most surgeons that a ganglion is essentially a protrusion of synovium through the tendon sheath which undergoes colloid degeneration. It has been held, however, to be caused by the colloid degeneration of fœtal relics in the synovium. This seems a little far-fetched.

The best treatment is excision. The old-time remedy for a ganglion was to beat it flat with a heavy object, generally a book, and subsequently to apply pressure to the part. This is a grossly unsurgical procedure, and should not be countenanced. Cases have been recorded in which an apparently simple ganglion was treated in this way, and the patient subsequently died from generalised tuberculosis. A very small percentage of apparently simple ganglia are in reality manifestations of tuberculosis, and to beat them with a book merely disseminates the toxins. It is allowable to withdraw the contents of the ganglion with an aspirating syringe and then apply pressure, if it is particularly desired to avoid a scar. A certain cure cannot be promised, thus, but at any rate, no serious damage is likely to ensue, even if the case be tuberculous. The only really satisfactory treatment is excision. This is not always as easy as might be thought. Very often the ganglion has a broad base, so that it is difficult to apply a ligature round it without rupturing it, and the moment one ruptures the swelling one's difficulties are greatly increased, because a clear translucent jelly-like material exudes, which renders the field of operation greasy. In such a case one may be compelled simply to snip off the protruded synovium, and leave it at that. But it is always better, if possible, to ligature the base of the ganglion before cutting it off.

A compound palmar ganglion is a much less simple affair. This is really an inflammatory condition of the common synovial sheath of the flexor tendons of the fingers. Two main forms are found; one in which the synovial cavity is distended with fluid, and the other in which the synovium itself is much thickened, and contains a number of so-called melon-seed bodies. These cases are nearly all tuberculous in origin, and there is really very little justification for dividing them into two classes. The condition is analogous to tuberculous disease of the synovial membrane of a joint. In this one may find an excessive secretion of fluid (hydrops articuli), or the synovium only may be thickened. As in joint disease so in this, the pulpy swelling is more common than the hydrops.

The patient complains of a swelling on the front of the wrist. In a typical case it is dumb-bell shaped, the central constriction corresponding to the position of the anterior annular ligament, which lies in front of the swollen synovial membrane and compresses it. If there is free fluid, fluctuation is communicated from the portion above to the portion below the anterior annular ligament. The melon-seed bodies, which are composed of altered fibrin, if numerous, occasionally cause a sensation of crepitus.

The treatment consists in making an incision into the swelling above and below the annular ligament and evacuating any fluid. A drainage tube is then inserted. This can be removed in a week, and the wounds allowed to heal. Some authorities have suggested the introduction of iodine into the sac with the object of setting up a low form of inflammation which will cause the walls of the cavity to adhere to each other. This is not to be advised, because, even if it is successful, it usually leads to impairment of the functions of the flexor tendons. In the case where the synovium is pulpy and thickened, it must be scraped, or, if possible, dissected away and constitutional treatment adopted, as for any other tuberculous lesion; but the prognosis is distinctly less favourable, since the joints, and even the bones of the carpus, may be infected; and it may become necessary eventually to amputate the hand.

